# The Role of *MAPT* Haplotype H2 and Isoform 1N/4R in Parkinsonism of Older Adults

**DOI:** 10.1371/journal.pone.0157452

**Published:** 2016-07-26

**Authors:** Guilherme T. Valenca, Gyan P. Srivastava, Jamary Oliveira-Filho, Charles C. White, Lei Yu, Julie A. Schneider, Aron S. Buchman, Joshua M. Shulman, David A. Bennett, Philip L. De Jager

**Affiliations:** 1 Movement Disorders Clinic, Roberto Santos General Hospital, Salvador, BA, Brazil; 2 Health Sciences Center, Federal University of Reconcavo of Bahia, Santo Antonio de Jesus, BA, Brazil; 3 Post-Graduate Program in Health Sciences, Federal University of Bahia, Salvador, BA, Brazil; 4 Program in Translational Neuropsychiatric Genomics, Departments of Neurology & Psychiatry, Brigham and Women’s Hospital, Boston, Massachusetts, United States of America; 5 Harvard Medical School, Boston, Massachusetts, United States of America; 6 Program in Medical and Population Genetics, Broad Institute, Cambridge, Massachusetts, United States of America; 7 Rush Alzheimer’s Disease Center, Rush University Medical Center, Chicago, Illinois, United States of America; 8 Departments of Neurology, Molecular and Human Genetics, and Neuroscience, and Program in Developmental Biology, Baylor College of Medicine, Houston, Texas, United States of America; 9 Jan and Dan Duncan Neurological Research Institute, Texas Children’s Hospital, Houston, Texas, United States of America; Cleveland Clnic Foundation, UNITED STATES

## Abstract

**Background and Objective:**

Recently, we have shown that the Parkinson’s disease (PD) susceptibility locus *MAPT* (microtubule associated protein tau) is associated with parkinsonism in older adults without a clinical diagnosis of PD. In this study, we investigated the relationship between parkinsonian signs and *MAPT* transcripts by assessing the effect of *MAPT* haplotypes on alternative splicing and expression levels of the most common isoforms in two prospective clinicopathologic studies of aging.

**Materials and Methods:**

using regression analysis, controlling for age, sex, study and neuropathology, we evaluated 976 subjects with clinical, genotyping and brain pathology data for haplotype analysis. For transcript analysis, we obtained *MAPT* gene and isoform-level expression from the dorsolateral prefrontal cortex for 505 of these subjects.

**Results:**

The *MAPT* H2 haplotype was associated with lower total *MAPT* expression (p = 1.2x10-14) and global parkinsonism at both study entry (p = 0.001) and proximate to death (p = 0.050). Specifically, haplotype H2 was primarily associated with bradykinesia in both assessments (p<0.001 and p = 0.008). *MAPT* total expression was associated with age and decreases linearly with advancing age (p<0.001). Analysing *MAPT* alternative splicing, the expression of 1N/4R isoform was inversely associated with global parkinsonism (p = 0.008) and bradykinesia (p = 0.008). Diminished 1N/4R isoform expression was also associated with H2 (p = 0.001).

**Conclusions:**

Overall, our results suggest that age and H2 are associated with higher parkinsonism score and decreased total *MAPT* RNA expression. Additionally, we found that H2 and parkinsonism are associated with altered expression levels of specific isoforms. These findings may contribute to the understanding of the association between *MAPT* locus and parkinsonism in elderly subjects and in some extent to age-related neurodegenerative diseases.

## Introduction

Parkinsonian signs, characterized by bradykinesia, rigidity, tremor and gait disturbance, are common in older individuals [1] and are associated with functional impairment, higher risk of cognitive decline, dementia and mortality [2]. Recently, several reports demonstrated an association between pathology in brainstem nuclei and cerebrovascular lesions with mild parkinsonism [3,4]. In previous work, we have shown that the Parkinson’s disease (PD) susceptibility locus *MAPT* is associated with mild parkinsonism in older adults without a clinical diagnosis of PD, but the biological mechanism underlying this association is unknown [5]. The *MAPT* gene lies on chromosome 17q21, and its encoded protein Tau is involved in microtubule stability and interaction with the cytoskeleton [6]. Six isoforms of this protein, resulting from alternative splicing of exons 2, 3 and 10, are expressed in the adult human brain [7]. In the region containing *MAPT*, a single nucleotide polymorphism (SNP) tags the two major haplotype clades, termed H1 and H2, which are defined by a large inversion containing *MAPT* as well as several other genes. Many studies in the past decade have shown that this locus affects disease risk. *MAPT* H1 and its sub haplotype H1c are reported to be associated with increased risk for certain age-related neurodegenerative diseases, including progressive supranuclear palsy (PSP) [8–11], corticobasal degeneration (CBD) [10,11], Multiple System Atrophy (MSA) [12] and Parkinson’s disease (PD) [13,14]. These nosological entities also share a clinical spectrum ranging from parkinsonism to dementia. The H2 haplotype is related in some studies to late onset Alzheimer’s disease and frontotemporal dementia risk [15,16]; it is also associated with lower brain MAPT expression levels in Alzheimer’s disease (AD) patients [15]. Moreover, *MAPT* alternative splicing and expression have been shown to be important in neuropathological processes [6,17]. Our study refines the role of this locus in age-related parkinsonism and examines the hypothesis that the role of the *MAPT* haplotypes in age-related parkinsonism in older adults from the Religious Orders Study [18] and Rush Memory Aging Project (MAP) [19] may be mediated, in part, by alternative splicing. Exploring the pathophysiology of mild parkinsonism in older individuals can lead us to a better understanding of motor phenotypes in age-related neurodegenerative diseases.

## Materials and Methods

### Subjects selection from Cohorts

All of the samples used in this project are from autopsied brains obtained from participants in two longitudinal studies of aging, the Religious Orders Study (ROS) [18], started in 1994, and Rush Memory and Aging Project (MAP) [19], started in 1997. Both studies were approved by the Institutional Review Board of Rush University Medical Center. All subjects were older and free of known dementia at the time of enrollment and were followed annually with detailed clinical evaluations with a signed informed consent and an Anatomical Gift Act for brain donation. In total, over 3,000 participants have enrolled and 1,200 autopsied to date. Of these, 976 brains were available for the present study.

### Genotyping

DNA was extracted from whole blood, lymphocytes, or frozen postmortem brain tissue. Genotyping was done in three subsets. Genotyping of the first two sets of samples were generated using Affymetrix GeneChip 6.0 platform in 2009 at the Broad Institute’s Center for Genotyping, and the third set of samples were genotyped on the Illumina HumanOmniExpress platform in 2012 at the Children’s Hospital of Philadelphia. All three datasets were processed through the same quality control analysis and imputation pipeline that has been published in detail previously [5,20]. In this study, we investigated the two major *MAPT* locus haplotypes by tagging the H1 haplotype and H2 haplotype with the major allele and minor allele of rs1052553 (MAF = 0.20), respectively. The subhaplotype H1c was tagged with the minor allele of SNP rs242557 (MAF = 0.38) [21].

### *MAPT* gene total expression and Isoforms-level expression using RNA-seq data

RNA-seq data was generated using postmortem brain tissue (dorsolateral prefrontal cortex) from ROS/MAP subjects. The RNA was extracted from the tissue after the quality control evaluation based on RNA Integrity Number (RIN score) [22]. The library was sequenced using Illumina Hi-Seq with 101bp reads with 4-plex pooling. All the paired-end reads were mapped using TopHat software [23] using the human genome transcriptomic database from Ensemble (http://www.ensembl.org). The Fragment Per Kilobase Per Million (FPKM) was calculated for each isoform of each gene from the human genome. These FPKM values were regarded as expression quantity for each gene and its isoform for further analysis.

### Clinical and pathological outcome measures

The parkinsonian symptoms measurements were performed annually using a 26-items modified version of the motor section of the Unified Parkinson Disease Rating Scale (mUPDRS). Four previously established parkinsonian sign scores (bradykinesia, rigidity, tremor, and gait impaiment) were derived from these 26 items, and a summary global parkinsonism score was constructed by averaging these 4 scores, as previously described (3). We analyzed the first and last measurements of these scores as separate continuous outcomes in multivariable models, but focused primarily on the last measurement because it was taken closest to the time of autopsy.

Postmortem assessment for Lewy bodies, neurofibrillary tangles, neuritic plaque and macro-microscopic cerebral infarcts were collected as detailed in prior publications [4, 24–26].

### Statistical Analysis

In models linking parkinsonism, and its elemental motor traits, with the *MAPT* haplotype and *MAPT* expression, parkinsonian outcomes (global parkinsonism, bradykinesia and gait impairment) were skewed to the right and square root transformations were applied prior to the analysis. Similarly, neuritic plaques and neurofibrillary tangles were square root transformed. We dichotomized the measurements of rigidity and tremor due to a excess of 0 values, where non-zero values were represented as one and 0 values were represented as 0 in order to increase the signal to noise ratio. In the analysis of *MAPT* expression, we used number of Fragments Per Kilobase of transcripts per Million mapped reads (FPKM values) to quantify the expression of each isoform and aggregated these FPKM values in order to quantify total gene expression. We summarize continuous variables with means and standard deviations, and dichotomous variables with percentages, for the analyzed population in Table 1.

**Table 1:** Cohort characteristics for 976 subjects used for the analysis.

Study Subject Characteristics	mean +/- SD or n(%)
**Demographics and Clinical Variables**	
Number of subjects	976
Age at death	88.36 ± (6.5)
Age at baseline visit	81.02 ± (6.91)
Education	16.44 ± (3.61)
Male	35.9% (351)
PD diagnosis at death	6.2% (60)
Subjects with RNA data	51.7% (505)
Percent in ROS (versus MAP)	53.2% (519)
**mUPDRS scores**	
Bradykinesia score baseline visit	3.31 ± (1.94)
Bradykinesia score last visit	3.79 ± (2.26)
Global parkinsonism score baseline visit	3.07 ± (1.26)
Global parkinsonism score last visit	3.95 ± (1.41)
Gait score baseline visit	4.02 ± (2.01)
Gait score last visit	5.78 ± (2.05)
Rigidity > 0 baseline visit	33.3% (324)
Tremor score > 0 baseline visit	45.8% (445)
Rigidity > 0 last visit	53.4% (517)
Tremor score > 0 last visit	48.4% (471)
**Post Mortem Pathology**	
Lewy bodies present in nigra	21.5% (210)
Neurofibrillary tangles	0.67 ± (0.42)
Neuritic plaques	0.74 ± (0.54)
Macroscopic infarcts	35.7% (348)
Microscopic infarcts	28.5% (278)

We applied linear regression for continuous outcomes (bradykinesia, gait and global parkinsonism), and logistic regression models for tremor and rigidity. All models testing for associations with parkinsonism were adjusted for pertinent covariates, including age, sex, study (ROS or MAP) and various brain pathologies, if available at time of measure, including Lewy body (presence or absence), microinfarct (presence or absence), macroinfarct (presence or absence), neuritic plaque and neurofibrillary tangles. In analysis testing association between haplotype and expression, age, sex, cohort and neuropathology were included as covariates in the models. Analyses were done using the base stats package in R version 3.1 (www.r-project.org).

## Results

For the analysis of haplotypes in relation to parkinsonism, a total of 976 subjects with non-missing genotype and phenotype data were evaluated. A subset of 505 of these subjects also had *MAPT* RNA sequencing data available (51.7%) (Table 1). Allele and genotype frequencies for *MAPT* rs1052553 and rs242557 are shown in S1 Table. The rs1052553 SNP tags the *MAPT* H1/H2 haplotypes, and the rs242557 SNP tags the *MAPT* H1c subhaplotype. As expected, advancing age is strongly associated with worsening motor traits (global parkinsonism: β = 0.040, p = 2.8x10-8; S2 Table).

### Association of *MAPT* haplotypes with parkinsonism and its four comprising components

**Fig 1:**
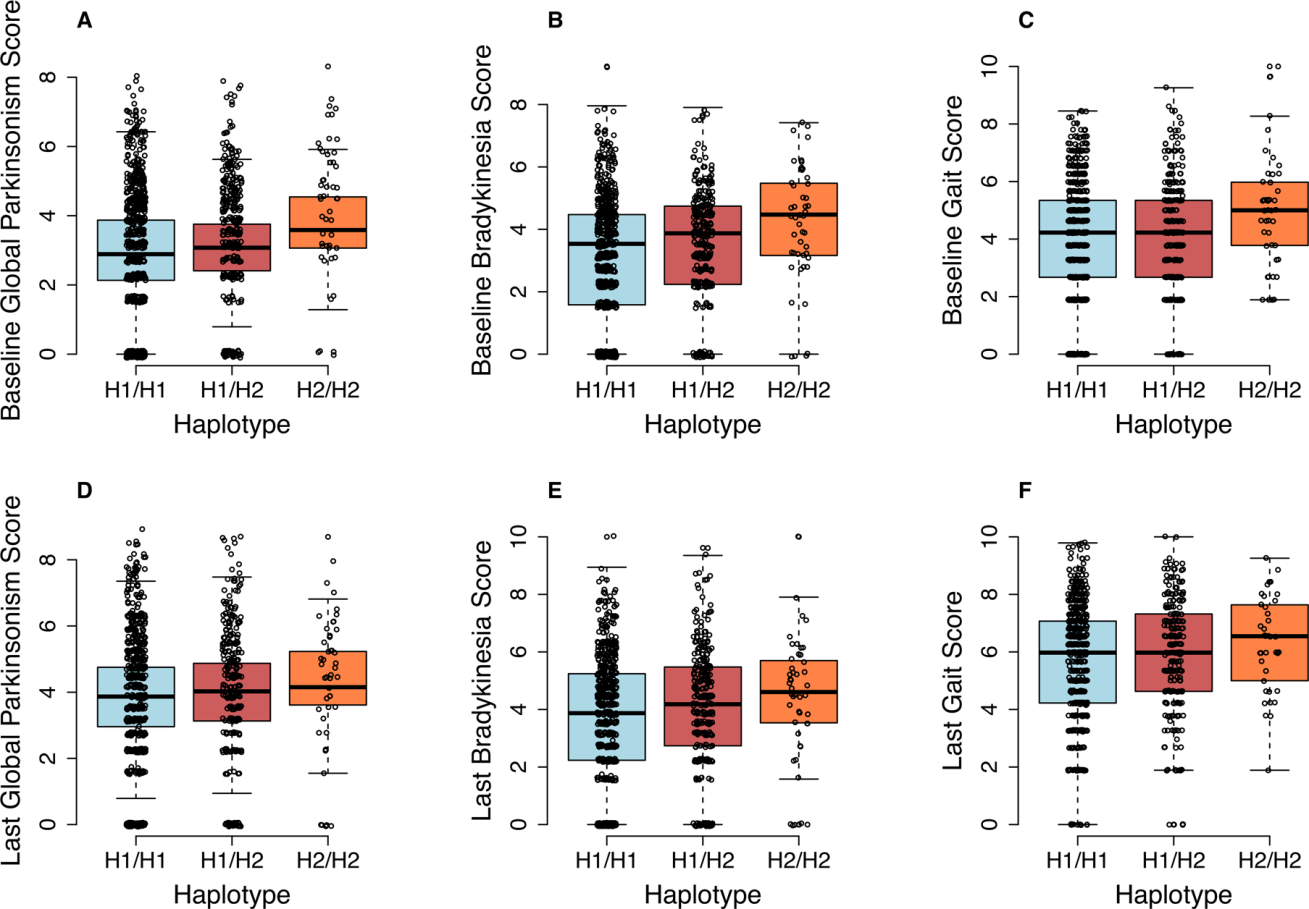
Relation of clinical signs to the H1-H2 haplotype. The tag SNP rs1052553 is used to differentiate the H1/H2 haplotypes. The top series of panels (A, B, C) report the association of MAPT genotype with motor traits at the baseline assessment (global parkinsonism, p = 0.001; bradykinesia, p<0.001; gait, p = 0.021, adjusted for age, sex, study). The bottom series of panels (C, D, E) report the association of MAPT genotype with motor traits at the time of the last available assessment (global parkinsonism, p = 0.050; bradykinesia, p = 0.008; gait, p = 0.12, adjusted for age, sex, study, + path). Each dot represents one subject.

Using an additive model, we examined the association between the two major *MAPT* haplotypes and global parkinsonism as well as its four component motor domains: bradykinesia, gait impairment, tremor and rigidity, which are measured at both at baseline and annually thereafter (Table 2)(Fig 1). In our data, we observed an association of the *MAPT* H2 haplotype with greater global parkinsonism and its component measures of bradykinesia and gait score, but not rigidity or tremor, at the baseline measurement of each subject. By contrast, the *MAPT* H1c haplotype is not associated with global parkinsonism or its component measures. Interestingly, the association between the H2 haplotype and global parkinsonim or gait is no longer detected when considering data obtained at the last evaluation proximate to death nd adjustment for measured neuropathologies (Table 2)(Fig 1), which may be many years after the baseline evaluation. However, the association with bradykinesia persists, with an effect size that is essentially unchanged (Table 2)(Fig 1).

**Table 2:** *MAPT* H1 and H2 major haplotypes* and *MAPT* subhaplotype H1c** association with global parkinsonism and motor components at baseline^a^ and last^b^ measurements.

Parkinsonism Component		rs1052553*		rs242557**	
		Estimate	Pvalue	Estimate	Pvalue
**Baseline measurement**					** **
** **	Global Parkinsonism	0.21	0.001	0.093	0.13
** **	Bradykinesia	0.37	<0.001	0.13	0.19
** **	Gait impairment	0.23	0.021	0.15	0.11
** **	Rigidity	0.12	0.31	0.15	0.17
** **	Tremor score	0.17	0.15	0.059	0.58
** **					
**Last measurement**					
** **	Global Parkinsonism	0.15	0.050	-0.015	0.82
** **	Bradykinesia	0.32	0.008	0.086	0.45
** **	Gait	0.18	0.12	-0.012	0.91
** **	Rigidity	0.15	0.20	-0.13	0.22
** **	Tremor	-0.10	0.38	-0.068	0.52

^a^Summary of association test using linear regression for global parkinsonism, bradykinesia and gait and logistic regression for dichotomized rigidity and tremor measurement, after adjustment for age,sex, and study.

^**b**^Summary of association test using linear regression for global parkinsonism, bradykinesia and gait and logistic regression for dichotomized rigidity and tremor measurement, after adjustment for age, sex, study, Lewy body, neuritic plaque, neurofibrillary tangle, macroscopic infarct and microscopic infarct.

These associations remained following exclusion of subjects with a PD diagnosis: global parkinsonism at baseline adjusting for age, sex, study (p<0.001), and p = 0.064 proximate to death adjusting for age at death, sex, study and pathology. Bradykinesia associations were also not significantly affected: p<0.001 at baseline adjusting for age, sex, study, and p = 0.019 proximate to death adjusting for age at death, sex, study and pathology.

In older age, other neuropathologies can also influence parkinsonian signs in this cohort, including macroscopic infarcts (β = 0.415, p<0.0001) and Lewy bodies (β = 0.27, p = 0.009) (S2 Table). Once these confounding effects are accounted for in the model, an association with global parkinsonism is seen at the last evaluation and appears to be driven primarily by an effect on bradykinesia (p = 0.008) (Table 2). These intriguing results suggest that the effect of the H2 haplotype on bradykinesia may be more pronounced earlier in life and that this effect is obscured in later life by the accumulation of neurological insults to the brain such as cerebral infarcts and Lewy bodies.

### Association of age, parkinsonian signs and the H1 and H2 haplotypes with total *MAPT* gene brain expression

**Fig 2:**
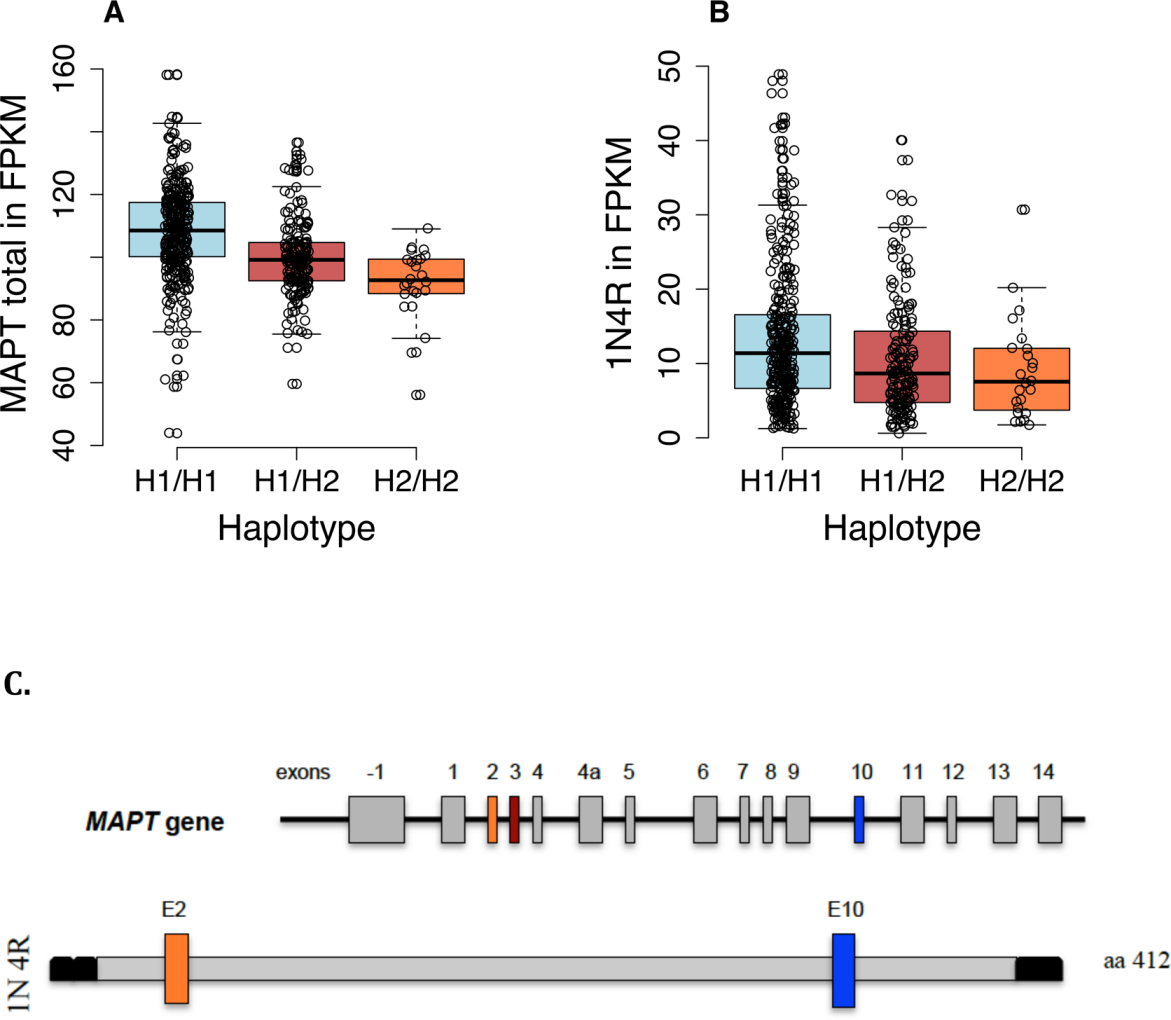
Relation of *MAPT* expression to the H1/H2 haplotypes. A dose-dependent effect of the H2 haplotype is noted. Each dot represents one subject. (A) Total *MAPT* expression (p = 1.2x10-14 adjusted for age, sex, and path); (B) Expression of *MAPT* isoform 1N4R, the only isoform to be significantly associated with parkinsonism. (p<0.001 adjusted for age, sex, and study and p = 0.001 adjusted for age, sex, study and path); (C) *MAPT* exon structure and composition of the 1N4R isoform: aa, amino acids; E2, exon 2; E3, exon 3; E10, exon 10.

In order to gain insight into the association between the *MAPT* haplotypes and parkinsonian signs, we further analyzed the relationships between the haplotypes, *MAPT* RNA expression, and parkinsonian phenotypes. First, we found that *MAPT* total mRNA expression is strongly influenced by age (p<0.001 age, sex, study and H2 only; p<0.0001 adjusting for age, sex, study, H2 and pathology), decreasing linearly with advancing age in all *MAPT* haplotype categories (S1A Fig). In the analysis testing for evidence of association between haplotypes and total *MAPT* expression, age, sex, study and neuropathology were included as covariates in the models. Our results show that rs1052553, the tag SNP differentiating the H1/H2 haplotypes, strongly influences *MAPT* total expression: as shown in Fig 2A, the H2 haplotype is strongly associated with lower *MAPT* total expression relative to the H1 haplotype (p = 1.2x10^-14^ adjusted for age, sex, and pathology). Although we observed increased global parkinsonism, bradykinesia and gait disturbance scores with decreasing *MAPT* gene total expression, the association between them was not significant (S2A Fig).

### *MAPT* H2 haplotype, motor traits and *MAPT* isoform level analysis

Since the literature suggests that altered levels or ratios of *MAPT* RNA isoforms play a role in the susceptibility of age-related neurodegenerative diseases associated with motor impairment [27], we also analysed the relationship between isoform levels to parkinsonism and *MAPT* haplotypes. Six different isoforms of *MAPT* are available in our RNA sequencing data. Adjusting for age, sex and brain pathology, we found that the level of the 1N/4R *MAPT* isoform was inversely associated with global parkinsonism (β = -0.0173; p = 0.008, adjusted for age, sex, study and pathology), bradykinesia (β = -0.0281; p = 0.008, adjusted for age, sex, study and pathology) and gait impairment (β = -0.019; p = 0.039) at the last visit prior to death. (S2B Fig). After further adjusting for *MAPT* total level of expression, this isoform remained associated with global parkinsonism (β = -0.0165, p = 0.012, adjusted for age, sex, study and pathology) and bradykinesia (β = -0.0276, p = 0.011, adjusted for age, sex, study and pathology) (S3 Fig). None of the other *MAPT* common isoforms expressed in the brain showed association with parkinsonian signs in a model controlling for total expression level and brain pathologies.

We next investigated the relation of the *MAPT* haplotypes to the level of this RNA isoform. Interestingly, the H2 haplotype was associated with lower expression of the 1N/4R isoform (p<0.001 adjusting for age, sex and study; p = 0.001 after adjustment for pathology) (Fig 2B). In addition, age was not associated with the MAPT 1N/4R (p = 0.58)(S1B Fig).

We also assessed whether the *MAPT* RNA 1N/4R isoform expression mediated the effect of the H2 haplotype on the clinical trait that is most strongly associated with it, bradykinesia. In this analysis, a reduction of effect size by >10% is taken as evidence of mediation. Here, we see that inclusion of the 1N/4R isoform in the analytic model diminishes the β for the H2 haplotype in relation to bradykinesia by 16%, suggesting that it may mediate, in part, the effect of the H2 haplotype on bradykinesia. We note that the effect of the 1N/4R isoform is reduced when total *MAPT* gene expression is added to the model (β decreases 3% with 1N/4R). Thus, while the effects on MAPT expression and splicing may mediate part of the H2 effect, most of the effects of H2 and 1N/4R on bradykinesia are independent, suggesting that they represent largely independent processes that influence aging-related functional decline (p value of 1N/4R = 0.017, p value of H2 = 0.012).

## Discussion

Parkinsonism, characterized by bradykinesia, rigidity, tremor, gait and balance problems, can commonly be identified during clinical examination of older individuals without prior diagnosis of neurological conditions^1^. These motor features are associated with mild cognitive impairment, increased risk of dementia, PD, depressive symptoms, and cerebrovascular lesions [28]. They are also a significant predictor of mortality [4,29]. Recently, we have identified two genetic variants implicated in PD susceptibility as risk factors for age-related motor impairment; one of these resides in the *MAPT* locus [5].

Multiple studies have reported the role of haplotype H1 and subhaplotype H1c as risk factors for age-related neurodegenerative diseases that share parkinsonian phenotypes, such as PD, MSA, PSP and CBD [8–14]. By contrast, in our cohorts, we observed a significant positive correlation between *MAPT* H2 haplotype and global parkinsonism. In fact, it is the bradykinetic component of parkinsonism, which is commonly seen in older individuals [2], that drives the association of parkinsonism with *MAPT* H2. We also found that the association between parkinsonism and H2 is stronger in our baseline measurements than in the measurement taken closest to death. This implies that H2 drives parkinsonism at a younger age and that its effect may wane over time. This observation also may be explained in part by the effect of other factors affecting the same trait, such as other neuropathologies (e.g., the presence of Lewy bodies and macroscopic infarcts), age and possibly epigenetic mechanisms [30,31]. Given that the mean age at death of our subjects is 88, the effect of H2 on parkinsonism might be significantly attenuated by the increasing prevalence of these other pathologies. In contrast with bradykinesia and gait, rigidity and tremor were not associated with H2 haplotype probably because these signs are not frequently observed in older people with parkinsonism [2]. These findings are consistent with our prior analysis in a subset of these subjects [5]. The subhaplotype H1c, tagged by the snp rs242557, which is implicated in tauopathy risk [11], was not associated with any of the parkinsonian measures. Unlike others studies that established the relationship between H1 and H1c and age-related neurodegenerative diseases, our results are based in two cohorts of older subjects free of these conditions. Therefore, the association between H2 haplotype and parkinsonism found in our study suggests different mechanisms to explain this phenotype in older individuals.

When we investigated the expression of total *MAPT* in 505 individuals, we observed that H2 was strongly associated with lower total *MAPT* expression compared when compared to H1. This result confirms a recent study [15] where the authors evaluated the association of *MAPT* haplotypes with brain *MAPT* gene expression levels in 202 late onset Alzheimer’s disease individuals. It was found that the H2 haplotype was associated with both lower *MAPT* RNA brain expression at the gene level and late onset AD risk [15]. We also observed that total *MAPT* brain expression decreases with age in each haplotype category. In fact, the decrease of total *MAPT* gene expression with advancing age has been described previously [32]. When placed in a single model, both age and the *MAPT* H2 haplotype are independently associated with lower total *MAPT* expression.

Another aspect of *MAPT* expression addressed in our study was the association of major haplotypes and parkinsonian measures with the six common *MAPT* isoforms. We observed that the levels of expression of only one of the *MAPT* isoforms expressed in the human brain were significantly associated with global parkinsonism and bradykinesia scores. Whilst 1N/4R isoform was inversely associated with motor findings, we found that H2 haplotype was associated with increased scores of global parkinsonism and bradykinesia, and lower expression of the transcript 1N/4R; therefore, the association of H2 on isoform expression results in competing effects on motor components. In addition, the mediation model suggests that the isoform and H2 haplotype influence these motor traits largely independently of one another. Interestingly, published data report that H2 haplotype subjects have 2-folder greater expression of isoforms with segments encoded by exons 2 and 3 [17,33]. Furthermore, Tau isoforms are released at differing rates depending on the contribution of the N terminus and microtubule binding repeat length [34,35]. This imbalanced expression of alternative transcripts is critical for neuronal function and may play a role in motor impairment in older people. Again, the advanced age of the subjects in our cohort could be a decisive factor for this imbalance and hence for this association [36,37].

In summary, our results begin to dissect the complex interplay of different risk factors related to the *MAPT* locus in its influence of aging-related parkinsonism, mainly through an effect that is manifested clinically as bradykinesia. In addition, we describe the relation of *MAPT* isoform expression in the frontal cortex in relation to the H1 and H2 haplotypes, providing additional data for understanding the role of this important locus in other neurologic diseases.

## Supporting Information

S1 Fig **(a) Relation between age at time of death and MAPT expression stratified by H1/H2 haplotype (p<0.001 age, sex, study and H2 only;** p<0.0001 adjusting for age, sex, study, H2 + path). (**b) Relation between age at time of death and MAPT 1N4R expression stratified by H1/H2 (p = 0.58 adjusting for pathologies).**
(PDF)

S2 Fig (a) Relationship between motor traits scores at time prior to death and MAPT total expression (global parkinsonism score, p = 0.14; bradykinesia score, p = 0.45; gait score, p = 0.12). (b) Relationship between motor traits scores at time prior to death and MAPT 1N4R total expression (global parkinsonism score, p = 0.008; bradykinesia score, p = 0.008; gait score, p = 0.039).
(PDF)

S3 Fig Relation between global parkinsonism score at time prior to death and MAPT isoform 1N/4R expression adjusted for MAPT total expression and brain pathology (p = 0.012). (PDF)

S1 Table Allele and genotype frequencies for *MAPT* rs1052553 and rs242557 based on 976 subjects. For rs1052553 allele A and G correspond to H1 and H2 haplotypes, respectively. For rs242557 allele A corresponds to the H1c haplotype.
(DOCX)

S2 Table Association of neuropathologies with global parkinsonism score at time of death. Neuropathologies, macroscopic infarcts and Lewy bodies, and age were also associated with global parkinsonism at time prior to death.Based on linear or logistic regression models.
(DOCX)
