# Supplementary material for: The Role of MAPT Haplotype H2 and Isoform 1N/4R in Parkinsonism of Older Adults
Source: PLoS One. 2016 Jul 26;11(7):e0157452. doi: 10.1371/journal.pone.0157452 (PMC4961370; doi:10.1371/journal.pone.0157452)

Figure S1a. Relation between age at time of death and *MAPT* expression stratified by H1/H2 haplotype (p<0.001 age, sex, study and H2 only; p<0.0001 adjusting for age, sex, study, H2 + path).

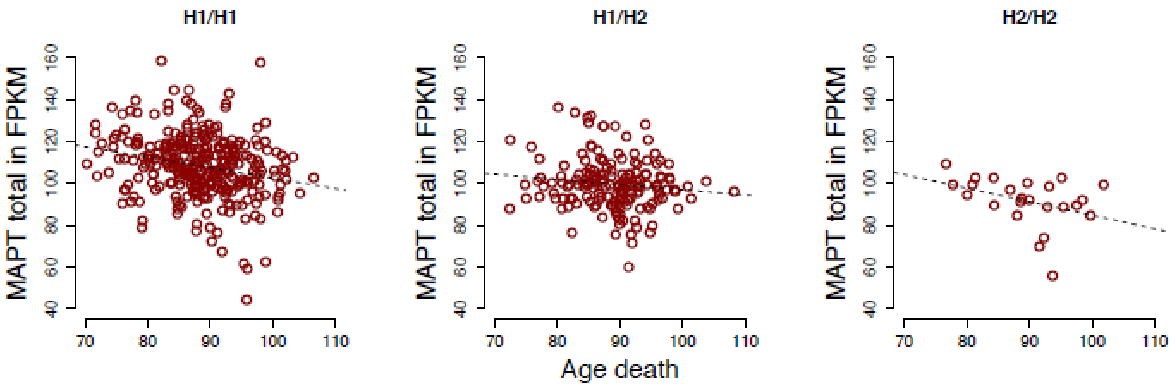

Figure S1b. Relation between age at time of death and *MAPT* 1N4R expression stratified by H1/H2 (p=0.58 adjusting for pathologies).

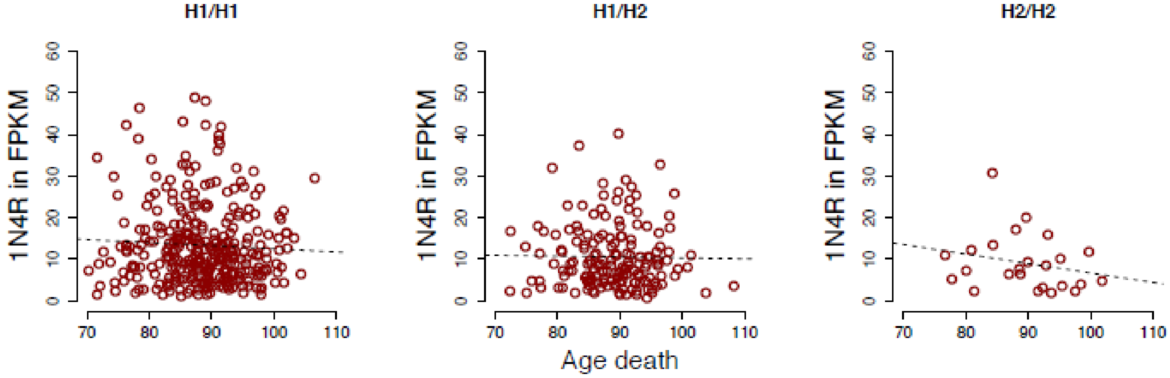

Supplement: S1 Fig — (a) Relation between age at time of death and MAPT expression stratified by H1/H2 haplotype (p<0.001 age, sex, study and H2 only; p<0.0001 adjusting for age, sex, study, H2 + path). (b) Relation between age at time of death and MAPT 1N4R expression stratified by H1/H2 (p = 0.58 adjusting for pathologies). (PDF) [file pone.0157452.s001.pdf]
