# Supplementary material for: The Role of MAPT Haplotype H2 and Isoform 1N/4R in Parkinsonism of Older Adults
Source: PLoS One. 2016 Jul 26;11(7):e0157452. doi: 10.1371/journal.pone.0157452 (PMC4961370; doi:10.1371/journal.pone.0157452)

Figure S2a. Relationship between motor traits scores at time prior to death and *MAPT* total expression (global parkinsonism score, p=0.14; bradykinesia score, p=0.45; gait score, p=0.12).

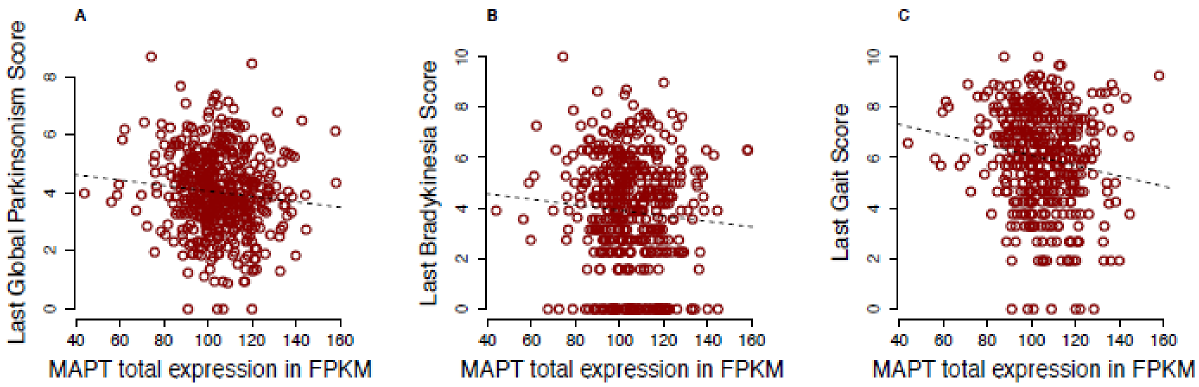

Figure S2b. Relationship between motor traits scores at time prior to death and *MAPT 1N4R* total expression (global parkinsonism score, p=0.008; bradykinesia score, p=0.008; gait score, p=0.039).

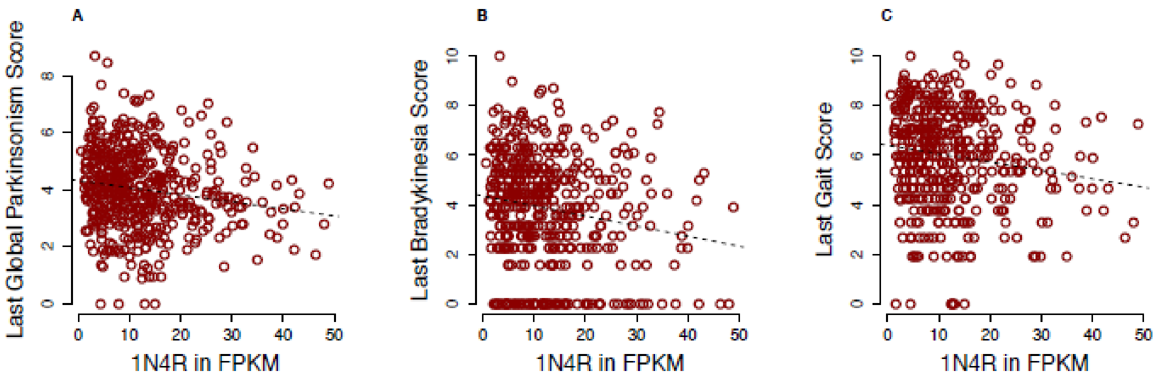

Supplement: S2 Fig — (a) Relationship between motor traits scores at time prior to death and MAPT total expression (global parkinsonism score, p = 0.14; bradykinesia score, p = 0.45; gait score, p = 0.12). (b) Relationship between motor traits scores at time prior to death and MAPT 1N4R total expression (global parkinsonism score, p = 0.008; bradykinesia score, p = 0.008; gait score, p = 0.039). (PDF) [file pone.0157452.s002.pdf]
