# Supplementary material for: The Role of MAPT Haplotype H2 and Isoform 1N/4R in Parkinsonism of Older Adults
Source: PLoS One. 2016 Jul 26;11(7):e0157452. doi: 10.1371/journal.pone.0157452 (PMC4961370; doi:10.1371/journal.pone.0157452)

Figure S3. Relation between global parkinsonism score at time prior to death and *MAPT* isoform 1N/4R expression adjusted for *MAPT* total expression and brain pathology (p=0.012).

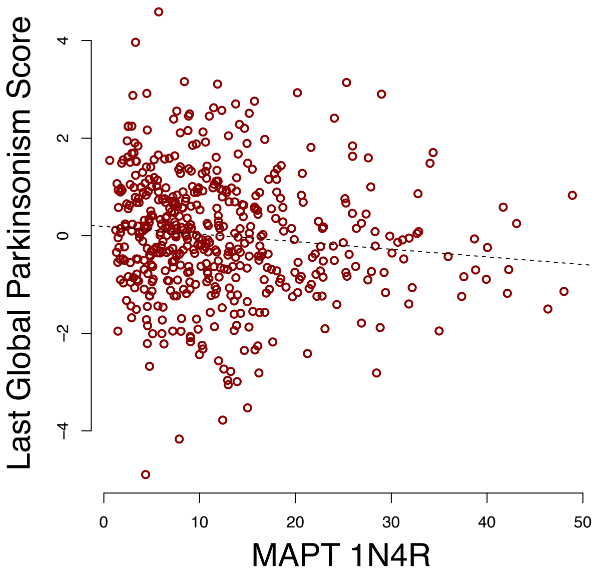

Supplement: S3 Fig — (PDF) [file pone.0157452.s003.pdf]
