# Supplementary material for: The Role of MAPT Haplotype H2 and Isoform 1N/4R in Parkinsonism of Older Adults
Source: PLoS One. 2016 Jul 26;11(7):e0157452. doi: 10.1371/journal.pone.0157452 (PMC4961370; doi:10.1371/journal.pone.0157452)
Supplement: S1 Table — For rs1052553 allele A and G correspond to H1 and H2 haplotypes, respectively. For rs242557 allele A corresponds to the H1c haplotype. (DOCX) [file pone.0157452.s004.docx]

**Table S1: Allele and genotype frequencies for *MAPT* rs1052553 and rs242557 based on 976 subjects**

| **SNP** | **Genotype, n (%)** | | | **Allele, n (%)** | |
| --- | --- | --- | --- | --- | --- |
|  | **AA** | **AG** | **GG** | **A** | **G** |
| rs1052553 | 631 (64.7%) | 298(30.5%) | 47 (4.8%) | 1560(80.0%) | 392(20.0%) |
| rs242557 | 103(10.6%) | 544(55.7%) | 329(33.7%) | 750(38.4%) | 1202(61.6%) |

For rs1052553 allele A and G correspond to H1 and H2 haplotypes, respectively. For rs242557 allele A corresponds to the H1c haplotype.
