# Supplementary material for: The Role of MAPT Haplotype H2 and Isoform 1N/4R in Parkinsonism of Older Adults
Source: PLoS One. 2016 Jul 26;11(7):e0157452. doi: 10.1371/journal.pone.0157452 (PMC4961370; doi:10.1371/journal.pone.0157452)
Supplement: S2 Table — Neuropathologies, macroscopic infarcts and Lewy bodies, and age were also associated with global parkinsonism at time prior to death.Based on linear or logistic regression models. (DOCX) [file pone.0157452.s005.docx]

**Table S2 . Association of neuropathologies with global parkinsonism score at time of death.**

| **Global Parkinsonism** | **Estimate** | **SE** | **P-value** |
| --- | --- | --- | --- |
| Intercept | -0.2 | 0.63 | 0.75 |
| rs1052553 | 0.15 | 0.074 | 0.050 |
| Age at death | 0.04 | 0.0071 | 2.8x10-8 |
| Male | -0.062 | 0.093 | 0.51 |
| Lewy bodies present in nigra | 0.27 | 0.11 | 0.009 |
| Neuritic plaques | 0.09 | 0.1 | 0.37 |
| Neurofibrillary tangles | 0.25 | 0.13 | 0.054 |
| Macroscopic infarcts | 0.42 | 0.095 | <0.0001 |
| Microscopic infarcts | 0.15 | 0.099 | 0.13 |
| Study | 0.24 | 0.088 | 0.006 |

Neuropathologies, macroscopic infarcts and Lewy bodies, and age were also associated with global parkinsonism at time prior to death.

Based on linear or logistic regression models.
